# A Rare Case of Esophageal Leukoplakia in Achalasia

**DOI:** 10.7759/cureus.23735

**Published:** 2022-04-01

**Authors:** Gowthami Kanagalingam, Yvette Achuo-Egbe, Mirza Fawad Ahmed, Oladimeji Oluaderounmu, Jennifer Harley

**Affiliations:** 1 Medicine, New York Medical College, Metropolitan Hospital Center, New York City, USA; 2 Gastroenterology and Hepatology, New York Medical College, Metropolitan Hospital Center, New York City, USA; 3 Gastroenterology, Metropolitan Hospital, New York City, USA

**Keywords:** acid reflux, disease surveillance, severe achalasia, esophageal leukoplakia, squamous cell carcinoma esophagus

## Abstract

Esophageal leukoplakia refers to a clinical finding of a white patch on the mucous membrane surface that cannot be scraped off. It has been associated with alcohol and tobacco use and chronic acid reflux. An association with squamous cell dysplasia and carcinoma has been reported with potential for malignant transformation warranting endoscopic intervention or surveillance, but no guidelines exist.

We present a case of a 77-year-old female with a history of longstanding achalasia requiring multiple Botox injections. After presenting with weight loss, esophageal dysphagia, and acid reflux the patient underwent an esophagogastroduodenoscopy (EGD) showing a 20 mm white plaque in the middle third of the esophagus and histopathology consistent with esophageal leukoplakia. After repeated Botox injection and treatment with PPI and H2 blocker, no findings of esophageal leukoplakia were noted on repeat EGD.

With this case, we aim to increase awareness of this rare disease pathology, especially in the setting of underlying achalasia. This case also raises the question if maximum anti-reflux therapy could have a potential benefit in avoiding the recurrence of esophageal leukoplakia.

## Introduction

Leukoplakia indicates a clinical term for a white plaque or patch on mucous membrane surfaces, which cannot be scraped off and is clinically not characterized as another disease.

Esophageal leukoplakia is a rare finding with six cases in 1,000 esophageal autopsy specimens or a prevalence of 0.19% among 1,048 esophageal biopsies. Esophageal leukoplakia is more common in middle-aged to elderly females and mostly affects the distal third part of the esophagus. Esophageal epidermoid metaplasia (EEM) is the histological correlate of the clinical finding of esophageal leukoplakia [1-3].

## Case presentation

We report on a 77-year-old female who has been following the gastroenterology clinic for achalasia. The patient has had multiple (EGDs) showing a narrowed lower esophageal sphincter (LES) requiring repeated Botox injections. EGD 10 years ago revealed a small segment of Barrett’s esophagus without dysplasia on histology. Regular surveillance for Barrett’s esophagus was continued for three years until the patient was lost to follow up.

The patient now presented to the clinic with 7-lb weight loss, esophageal dysphagia, worsening acid reflux, and regurgitation. Physical exam and laboratory results were unremarkable. A modified barium esophagogram was consistent with persistent narrowing of the thoracic esophagus near the gastroesophageal junction and marked dilation of the proximal thoracic esophagus due to achalasia.

**Figure 1:**
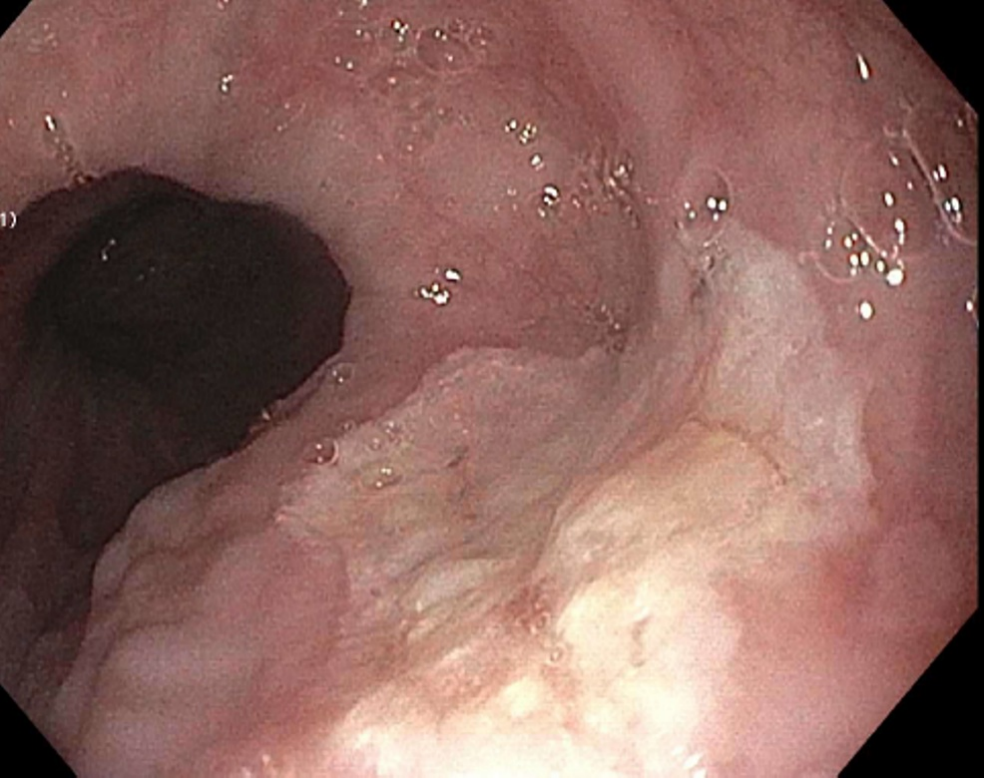
Esophageal leukoplakia: raised, well-demarcated, and oval-shaped white plaque in the middle third of the esophagus with normal surrounding mucosa

The patient then underwent EGD, which was consistent with narrowing of the gastroesophageal junction and erythematous stomach mucosa. A 20-mm white plaque in the middle third of the esophagus was noted. It was raised, well-demarcated, and oval-shaped with normal surrounding mucosa (Figure 1).

**Figure 2:**
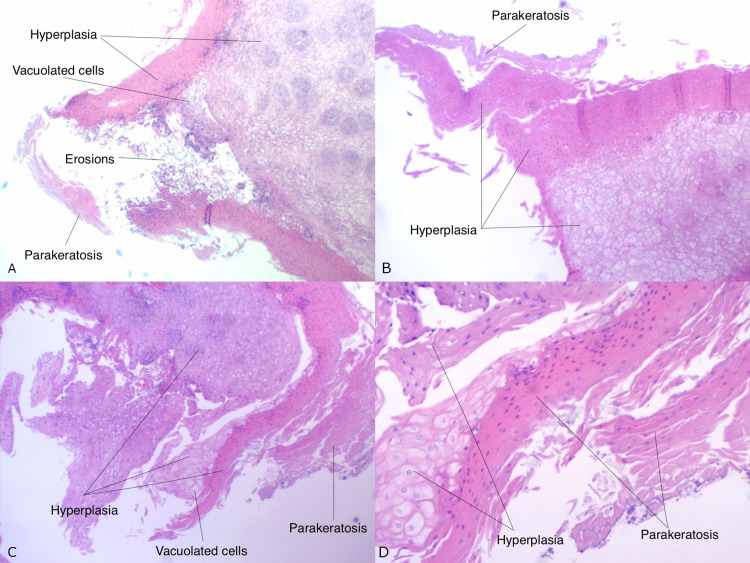
(A-D) Histopathology with epithelial hyperplasia, parakeratosis, vacuolated cells, surface erosion and inflammation

Histopathology confirmed the diagnosis of esophageal leukoplakia with epithelial hyperplasia, parakeratosis, surface erosion, and inflammation. Silver methenamine and PAS stains were negative for fungi but gram stain was positive for gram positive cocci. No goblet cells were found (Figures 2A-2D).

The patient refused surgical fundoplication. She underwent repeated Botox injections and was initiated on a twice daily PPI and H2 blocker therapy with improvement of her symptoms of dysphagia and acid reflux. Follow-up EGD six months later showed a dilated and tortuous esophagus with improved GE junction narrowing and short segment Barrett’s without dysplasia or malignancy. No lesions of esophageal leukoplakia were noted.

## Discussion

Esophageal leukoplakia is poorly described in the literature. It has only been reported in case reports or small case series [3,4].

The exact etiology of esophageal leukoplakia is unknown [5]. Association with alcohol consumption, tobacco smoking, or response to chronic acid reflux have been reported [6]. Further, esophageal dysmotility or stenosis causing chronic irritation have also been suggested as inciting factors [3]. Limido et al. reported a case of one patient with achalasia of the lower third of the esophagus and leukoplakia [7]. Similarly, our patient was a nonsmoker with no excessive alcohol consumption, leaving us with chronic irritation in the setting of achalasia as the possible cause of esophageal leukoplakia. This would also explain the disappearance of the lesions on EGD after repeated Botox injections and improvement of her acid reflux.

Esophageal leukoplakia is typically an incidental finding. Patients are usually asymptomatic but can present with globus sensation or dysphagia [5,6]. Endoscopic features include elevated scaly or white plaque, clearly demarcated borders from the surrounding normal tissue, a cobblestone or shaggy surface, and translucent white color. Endoscopic differential diagnoses of plaque-forming lesions includes esophageal papilloma, glycogenic acanthosis, infections such as Candida esophagitis, plaques related to acid reflux, or superficial esophageal cancer. Characteristic histological findings of esophageal leukoplakia include acanthosis, hyperplasia, and superficial esophageal squamous mucosa parakeratosis. A key histopathologic feature of esophageal leukoplakia is a marked granular layer with an overlying hyperorthokeratotic layer, similar to the epidermis of the skin [3,5].

Esophageal leukoplakia is often detected in close proximity to high-grade squamous cell dysplasia and squamous cell carcinoma suggesting it to be a preneoplastic lesion [1]. In a case series by Kamboj et al. with 40 patients with EEM 25% of the patients were found to have squamous cell neoplasia before, synchronous to, or after epidermoid metaplasia of the esophagus [8]. Singhi et al. reported a case series with 18 patients with esophageal leukoplakia amongst which 17% had high-grade dysplasia or even squamous cell carcinoma [1]. A small series by Ezoe et al. found metachronous or synchronous squamous cell carcinoma of the oropharynx and esophagus amongst four patients with EEM [9]. Interestingly, our patient had been diagnosed with short-segment Barrett’s esophagus and squamous hyperplasia several years before the diagnosis of esophageal leukoplakia. It is unclear if esophageal leukoplakia was already existing but small at the time Barrett’s esophagus was diagnosed or if this was an independent finding in a patient with underlying achalasia as a risk factor for Barrett’s esophagus.

No guidelines exist for the ideal frequency and pattern for evaluation or surveillance of esophageal leukoplakia [10]. Given concern for preneoplastic potential and its association with squamous cell dysplasia and esophageal cancer, the need for close surveillance or endoscopic interventions has been suggested [1,8]. Endoscopic resections for small dysplastic lesions and ablation therapy for large dysplastic lesions have been proposed. Further on, follow up with endoscopic targeted quadrantic biopsies every 1-2 cm done every six months followed by annual surveillance if no dysplasia or progression is seen for diffuse panesophageal or non resectable lesions without dysplasia were suggested [8].

Fortunately, our patient was not found to have any lesions of esophageal leukoplakia on repeat EGD, after maximal acid suppression therapy. This raises the question if acid suppression therapy could be a potential treatment option to avoid recurrence of esophageal leukoplakia.

## Conclusions

This is the second case report of a patient with underlying achalasia, diagnosed with esophageal leukoplakia. We aim to increase awareness of this relatively rare disease pathology. Due to its premalignant potential, close surveillance is necessary. Additional studies are needed to decide on the treatment, including potential acid suppression therapy and surveillance patterns for these patients, especially in patients with known risk factors, such as achalasia.
